# Systematic review on the effects of drug-coated balloon therapy on plaque morphology in patients with acute coronary syndrome

**DOI:** 10.1016/j.ahjo.2026.100844

**Published:** 2026-07-28

**Authors:** Kavithra Harihararajah, Zaynah Nawaz, Laiba Qadir, Shakil Ahmad, Derek Connolly, Irundika H.K. Dias, Mohammed Shamim Rahman

**Affiliations:** a Aston Medical School, College of Health and Life Sciences, Aston University, Birmingham, B47ET, UK; b Midland Metropolitan University Hospital and Birmingham City Hospital, Sandwell and West Birmingham NHS Trust Birmingham, West Midlands, UK

**Keywords:** Drug-coated balloons, Acute coronary syndrome, Plaque burden, Plaque volume, Plaque area, Intravascular imaging

## Abstract

**Background:**

In the treatment of diseased coronary arteries, drug-coated balloons (DCBs) have emerged as a “leave nothing behind” alternative to drug-eluting stents, yet their effects on coronary plaque morphology in Acute Coronary Syndromes (ACS) are not well characterised. This systematic review evaluates the impact of DCB therapy on plaque morphology in patients presenting with ACS.

**Method:**

A systematic search of PubMed, Cochrane Library, Scopus, and ScienceDirect identified studies reporting plaque-related outcomes following DCB treatment in adult patients with ACS. Six studies met the inclusion criteria, incorporating intravascular ultrasound (IVUS), optical coherence tomography (OCT), near-infrared spectroscopy (NIRS) or angiographic assessments of plaque burden, plaque volume, plaque area, lipid burden, or calcification.

**Results:**

Across comparative and non-comparative studies, DCB therapy was consistently associated with favourable imaging changes, including reductions in plaque area, plaque burden, or plaque atheroma volume, and increased lumen dimensions. In studies assessing compositional characteristics, DCB therapy was linked with reductions in lipid core burden and lower calcification scores compared with stent-based strategies. However, heterogeneity in study design, imaging methods, and outcome reporting limits direct comparison between studies, and causality cannot be conclusively inferred.

**Conclusion:**

Available evidence suggests that DCB therapy may favourably modify plaque morphology in ACS, influencing plaque size and composition. The potential for DCBs to promote plaque regression, reduce lipid burden, and facilitate positive vascular remodelling warrants further investigation in larger, dedicated ACS cohorts. Future studies integrating advanced imaging and molecular profiling may clarify mechanistic pathways and identify patient groups most likely to benefit from DCB-based strategies.

## Highlights

• Drug-coated balloon therapy was linked to favourable changes in plaque-related imaging parameters in ACS.
• Reported effects included reductions in plaque area, plaque burden, plaque volume, and lipid-rich plaque characteristics.
• In comparative studies, drug-coated balloons showed plaque-modifying effects broadly comparable to drug-eluting stents.
• Current evidence is limited and heterogeneous, supporting the need for larger imaging-based studies in ACS.

## 1 Introduction

Acute coronary syndrome (ACS) is a serious clinical condition that encompasses ST-segment elevation myocardial infarction (STEMI), non-ST-segment elevation myocardial infarction (NSTEMI), and unstable angina (NICE guideline, NG185, 2020) and is triggered by the disruption of atherosclerotic coronary plaques. Atherosclerotic plaque is composed of lipid-rich material, inflammatory cells, fibrous tissue, and variable calcification [1]. Plaque rupture may lead to adverse cardiovascular events, such as myocardial infarction (MI) or stroke. Plaque may be considered vulnerable when characterised by a thin fibrous cap and a large lipid-rich necrotic core [2]. These plaques are at significantly higher risk of rupture, triggering thrombus formation that may obstruct the coronary artery and result in MI or ischaemia [3].

Patients presenting STEMI require urgent treatment, as this is commonly associated with complete coronary artery occlusion and ongoing myocardial ischaemia [4]. Immediate reperfusion is essential to minimise myocardial damage. Studies have reported higher in-hospital mortality in STEMI compared with NSTEMI patients (5.4% vs 2.7%), who typically have partial coronary artery occlusion [5]. Although NSTEMI patients do not present ST-segment elevation, it may still cause significant myocardial injury and adverse long-term outcomes. Urgent revascularisation may be necessary in cases including cardiac failure, arrhythmia, or worsening myocardial ischaemia and chest pain [6]. Patients with unstable angina experience reduced coronary blood flow, causing chest pain at rest or on minimal exertion in the absence of detectable myocardial necrosis through biomarker testing. Despite this, unstable angina carries a high short-term risk of progression to MI. It is also considered part of the ACS spectrum and requires urgent medical assessment and hospital admission [7].

Significant coronary artery stenosis is commonly identified through invasive coronary angiography. In STEMI, rapid restoration of coronary blood flow is prioritised to minimise myocardial damage, where administration of aspirin (unless contraindicated) inhibits platelet aggregation and reduces thrombus formation. Additionally, in many ACS pathways, a second antiplatelet agent is administered, with choice influenced by bleeding risk and revascularisation strategy [8].

Percutaneous Coronary Intervention (PCI) is the gold-standard revascularisation procedure in most ACS cases, particularly in STEMI where rapid reperfusion through primary PCI is required. PCI is a minimally invasive technique used to open occluded or partially occluded coronary arteries, usually performed using balloon angioplasty followed by stent implantation [9]. Stent implantation is widely recognised as the standard PCI strategy for patients with coronary artery disease to reduce recoil and restenosis [10]. A stent is inserted into the diseased vessel to scaffold the artery and maintain luminal patency [11].

However, stent-based PCI leads to a permanent, intracoronary implant, which can cause in-stent restenosis (ISR), making management challenging. Placing an additional stent may increase metal burden and contribute to recurrent ISR [12]. Dual antiplatelet therapy (DAPT) is typically required after stent implantation, with duration tailored to individual ischaemic and bleeding risk. This may be problematic in patients with high-risk bleeding (HBR), where stent-based PCI may not always be suitable [13]. DCB-based approaches may reduce implant-related risk and permit shorter durations of DAPT, potentially lowering bleeding risk [14].

DCBs are coated with an antiproliferative drug such as paclitaxel or sirolimus. DCBs deliver these drugs directly to the vessel wall during balloon inflation, inhibiting vascular smooth muscle cell proliferation and reducing neointimal hyperplasia (NIH) [15]. NIH is a key mechanism underlying restenosis following coronary intervention with stent implantation. Neoatherosclerosis, the formation of new atherosclerotic plaque within stented areas, is another important cause of ISR, although it generally occurs later than NIH following stent implantation [16].

DCBs represent a promising alternative revascularisation strategy in ACS and may be associated with fewer device-related adverse effects [17]. Emerging evidence suggests that DCB therapy may influence coronary plaque morphology. However, their clinical significance in ACS patients remains poorly understood. Therefore, this systematic review aims to evaluate the effect of DCBs on plaque morphology. It focuses on plaque volume, burden and area using intravascular imaging modalities including intravascular ultrasound (IVUS), optical coherence tomography (OCT), near-infrared spectroscopy (NIRS), or quantitative coronary angiography (QCA). Both comparative and non-comparative studies are included to explore the potential effects of DCB therapy on plaque characteristics, without implying causality where study design does not support causal inference.

## 2 Methods

### 2.1 Literature search strategy

Literature was identified by searching PubMed, Cochrane Library, Scopus, and ScienceDirect. The search strategy used terms related to angioplasty, drug-coated balloon therapy, acute coronary syndrome, and coronary plaque. These search terms were aligned with Medical Subject Headings (MeSH) developed by the National Library of Medicine. The following search string was used: (“drug-coated balloon”) AND (“acute coronary syndrome”) AND (“plaque”). Articles were excluded if they did not report plaque-related outcomes following DCB therapy in the relevant population. The search was limited to studies published in English, with no restriction on publication year. Reference lists of relevant articles were also screened to identify additional studies.

### 2.2 Selection criteria

Studies included in this review were required to meet specific eligibility criteria. The population consisted of adult patients (≥18 years) with coronary artery disease, including STEMI, NSTEMI, or unstable angina. The intervention of focus was DCB therapy performed in the coronary arteries. Studies were included regardless of comparators, such as plain old balloon angioplasty or drug-eluting stents (DES).

The primary outcome assessed was coronary plaque morphology, measured using IVUS, OCT, NIRS, or QCA. This could be reported as plaque burden, plaque volume or plaque area. Studies reporting changes in plaque characteristics, such as calcification scores or lipid burden were also eligible. Where direct plaque measurements were unavailable, vessel characteristics such as lumen size or lumen enlargement were considered. Studies providing qualitative descriptions of plaque morphology without quantitative analysis were included.

Eligible study designs included randomised controlled trials, cohort studies, case series, and case reports.

### 2.3 Article selection

Two reviewers independently screened the titles and abstracts of all records identified through the search strategy described above. Duplicate records were removed, and review articles were excluded at this stage. The remaining articles were assessed for eligibility using the predefined inclusion criteria. Any disagreements were resolved through discussion or, where necessary, by consulting a third reviewer or principal investigator.

### 2.4 Study registration

The review protocol was registered with the International Prospective Register of Systematic Reviews database (PROSPERO), with a registration number PROSPERO 2025 CRD420251013074. This review was conducted in accordance with the Joanna Briggs Institute (JBI) Manual for Evidence Synthesis and reported in compliance with the Preferred Reporting Items for Systematic Review and Meta-Analysis (PRISMA) guidelines and checklist. The methodological quality of the included studies was assessed using the CASP (Critical Appraisal Skills Programme) checklist.

### 2.5 Data extraction

**Fig. 1:**
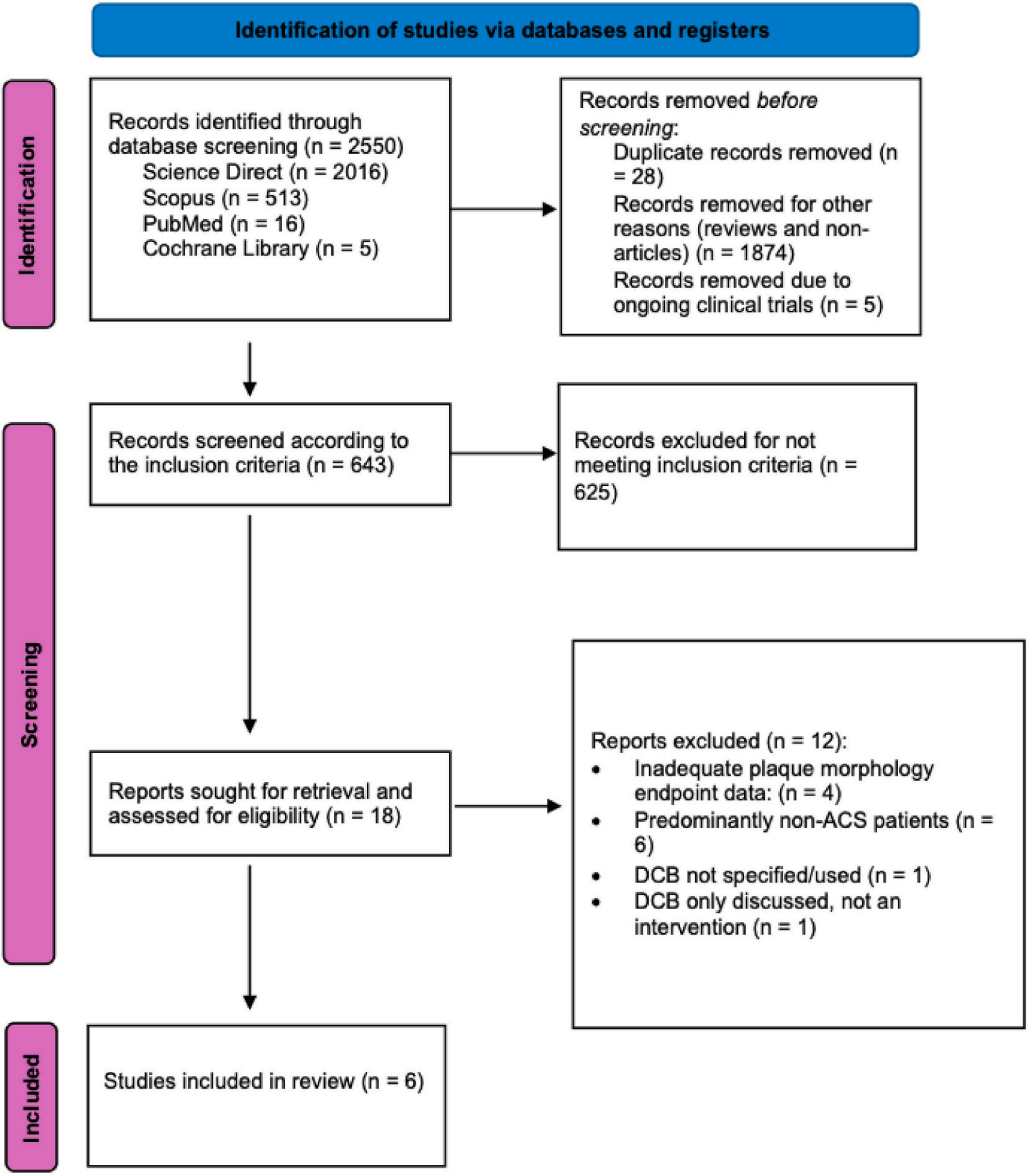
PRISMA Diagram: 2550 publications identified through the literature search, 18 were selected for full-text review and 6 were included in the analysis.

The initial search yielded 2550 records across the selected databases. After duplicate removal and title and abstract screening, potentially eligible studies underwent full-text review. Following screening, six studies met the predefined inclusion criteria and were included in the final analysis (Table 1) (Fig. 1, Table 2).

**Table 1:** Summary of findings across selected articles.

Author & Year of Publication	Study type and duration	Duration (months)	Study population	Number of Individuals (DCB treatment)	Age & Gender
Naohiro Funayama et al. 2021	Retrospective single-centre study	12	Patients with de-novo coronary artery lesions who underwent DCB angioplasty without stent implantation.	40	70.9 ± 7.8 Male: 32 Female: 8
Yu-Bin Zhang et al. 2022	Retrospective study	18	Patients diagnosed with ACS and given Percutaneous Coronary PCI.	55	56.40 ± 10.24 Male: 37 Female: 18
Ryota Fukuoka et al. 2024	Single-centre, prospective, single-arm study	6	Patients diagnosed with ACS who were undergoing emergency coronary angiogram.	30	69.1 ± 13.0 Male: 24 Female: 6
Anna van Veelen et al. 2024	Proof-of-concept, investigator-initiated, single-centre, prospective single-arm interventional trial	21	Patients presenting with non-ST-elevation acute coronary syndrome NSTE-ACS.	45	66 (55–73) Male: 18 Female: 27
Teruo Sekimoto et al. 2023	Case report	NA	Patient with a history of anxiety disorder and admitted with worsening chest pain. Diagnosed with anterior non-ST-elevation myocardial infarction.	1	42-year-old female
Kota Yamada MD et al. 2023	Single-centre retrospective study	48	Patients who underwent elective procedures for de novo coronary lesions.	275	68.5 ± 11.1 Male: 232 Female:43

**Table 2:** Summary of the observed effects of DCB therapy on coronary plaque across comparative and non-comparative studies.

Author	Type of Drug-Coated Balloon used	Imaging modality used	Comparative Study?	Observed effects on plaque (DCB only)
Naohiro Funayama et al. 2021	Paclitaxel-coated balloon (SeQuent Please, B. Braun, Malsungen AG, Germany).	IVUS	Yes ACS Vs SAP	Median plaque was assessed- DCB treatment resulted in a reduction of mean plaque area from 7.31mm^2^ pre-operatively to 7.21mm^2^ post procedure, with 6.9mm^2^ at the follow-up. Plaque burden data also revealed a 60% to 55% decrease post procedure- finally resulting in 50.1% plaque burden in the follow up.
Yu-Bin Zhang et al. 2022	Paclitaxel/Iohexol matrix coating on the Bingo DCB (Bingo™, Yinyi Biotech, Dalian, China)	Coronary angiography, IVUS	Yes DCB Vs DES	DCB treatment resulted in a reduction of plaque burden from 79.05 ± 4.17 preoperatively to 61.69 ± 5.64 post-procedure.
Ryota Fukuoka et al. 2024	SeQuent Please paclitaxel-coated balloon, Nipro	Coronary angiography, IVUS, OCT	Yes ‘bailout stent group’ Vs ‘RYUSEI DCB group’	Plaque atheroma volume and calcium score was assessed in this study: Plaque atheroma volume for Bailout Stent: 73.2 ± 0.85 Plaque atheroma volume for Ryusei DCB 67.7 ± 8.7 Calcium score for Bailout Stent: 24 ± 14 Calcium score for Ryusei DCB: 17 ± 9.2
Anna van Veelen et al. 2024	SeQuent Please NEO [B. Braun]	IVUS-NIRS	No	Plaque burden was assessed in this study: Plaque burden: DCB treatment resulted in a 47% to 44% reduction at 9 months follow-up
Teruo Sekimoto et al. 2023	DCB (SeQuent Please™, B. Braun Melsungen AG, Berlin, Germany)	OCT, IVUS	No	Thematic analysis of Plaque Burden given in this study: DCB treatment resulted in significant reduction in plaque burden.
Kota Yamada MD et al. 2023	SeQuent Please, B. Braun, Melsungen, Germany	IVUS	Yes DCB Vs DES	Thematic analysis of plaque mentioned in this study: Distribution of paclitaxel suppresses the plaque and induces the plaque regression.

## 3 Results

Most patients with coronary artery disease presented with ACS, a key focus of this review and search strategy. Although a small proportion had stable angina, it was used as a comparator in some studies. Four articles were comparative studies in which DCB therapy was compared with another PCI strategy, most commonly DES. The remaining two studies were non-comparative.

A case report by Sekimoto et al. 2023 provided qualitative intracoronary imaging descriptions of plaque-related findings. This report involved a 42-year-old woman with anterior non-ST-elevation myocardial infarction, presenting with worsening chest pain. OCT revealed healed plaques, while IVUS demonstrated “high-intensity marginal irregular masses at the culprit site”. In this report, lesion modification was performed using Excimer Laser Coronary Angiography (ELCA; Royal Philips) prior to DCB treatment. ELCA uses pulsed ultraviolet laser energy to modify plaque and coronary lesions. Following treatment with a 3.0 × 20 mm drug-coated balloon, OCT imaging revealed a lumen area of 5.49mm^2^, compared with 0.87mm^2^ at baseline. Although plaque burden or plaque volume were not quantitatively analysed, lumen area increased following ELCA and DCB. However, these findings cannot be attributed solely to DCB therapy because ELCA was also performed [18].

The second non-comparative study by van Veelen et al. 2024 screened 65 patients; 20 were enrolled to receive DCB treatment. The prophylactic paclitaxel-coated balloon (PCB) strategy was considered safe and feasible. Using IVUS-NIRS imaging, this study demonstrated a significant reduction in maxLCB_4mm,_ (maximum lipid burden found in any 4 mm segment at 9-month follow-up, suggesting a reduced lipid-rich plaque content). However, IVUS measurements showed no statistically significant change in overall plaque burden, which decreased from 47% at baseline to 44% at 9 months [19].

The remaining four comparative studies evaluated DCB therapy against DES across ACS and stable angina presentations. In the retrospective study conducted by Funayama et al. 2021, 40 coronary lesions were analysed; 27 patients with stable angina pectoris and 17 patients with ACS. IVUS findings demonstrated a significant reduction in median plaque area following DCB treatment in both ACS and stable angina groups. Plaque area decreased from 7.31 mm^2^ pre-procedure to 7.21 mm^2^ post-procedure, with a further reduction to 6.90mm^2^ at follow-up. IVUS-derived plaque burden decreased from 60.0% pre-procedure to 55.0% immediately post-procedure and to 50.1% at follow-up. These findings suggest reduced plaque burden following DCB treatment in both ACS and stable angina patients. However, observed changes were also associated with a significant increase in lumen area. Therefore, changes in plaque measurements may partly reflect vessel expansion. Furthermore, plaque area reduction was more pronounced in ACS patients compared with stable angina patients [20].

In the study conducted by Yu-Bin Zhang et al. 2022, DCB therapy was evaluated for safety and efficacy in ACS, particularly in lesions with vulnerable plaque characteristics. A total of 123 ACS patients were assigned to either DCB treatment (*n* = 55) or DES implantation (*n* = 68). IVUS analysis demonstrated a reduction in plaque burden from 79.05 ± 4.17% pre-procedure to 61.69 ± 5.64% post-procedure. A similar reduction was observed in the DES group, (80.07 ± 4.68% to 63.32 ± 6.07%). However, no statistically significant difference in plaque burden reduction was observed between treatment groups, suggesting comparable plaque-modifying effects between DCB and DES strategies [21].

A retrospective study was carried out by Yamada et al. 2023 analysed 588 consecutive coronary lesions, treated using either DCB (*n* = 275) or DES (*n* = 313). Qualitative analysis suggested that paclitaxel delivery from the DCB may suppress plaque progression and promote plaque regression. Furthermore, late lumen loss in the DCB group (0.04 ± 0.47 mm) was significantly lower than in the DES group (0.35 ± 0.76 mm; *p* < 0.001). This suggests that DCB treatment may better preserve lumen diameter over time [22].

The study conducted by Fukuoka et al. 2024 was a single-arm prospective study involving 30 patients treated with the Ryusei DCB and compared with a bailout stent group. The bailout stent group included patients requiring stent implantation after attempted DCB-based PCI due to vessel dissection or abrupt vessel closure. IVUS findings demonstrated that the bailout stent group exhibited greater plaque volume and higher calcification scores compared with the Ryusei DCB group. For example, plaque atheroma volume was 73.2 ± 0.85 mm^3^ in the bailout stent group compared with 67.7 ± 8.7 mm^3^ in the Ryusei DCB group. Calcium scores were higher in the bailout stent group (24 ± 14 Agaston Units.) compared with the Ryusei DCB group (17 ± 9.2 Agaston Units.) OCT imaging also demonstrated plaque rupture following DCB treatment, indicating lesion modification during the procedure. Lower calcification burden in the DCB group may reflect more favourable lesion characteristics for balloon-based therapy [23].

## 4 Discussion

DCB therapy was associated with changes in imaging-derived plaque characteristics, including plaque size, lumen dimensions, and composition, although substantial heterogeneity existed between studies. Furthermore, in studies where only thematic or qualitative analyses were performed, additional plaque-related characteristics were described. For instance, in Sekimoto et al. 2023, although the study did not quantitatively assess plaque burden or plaque volume, the increase in lumen area ELCA-assisted DCB treatment provides supportive imaging evidence of effective lesion modification [18]. An increase in lumen area is often associated with reduced plaque-related obstruction, suggesting that DCB therapy may be effective when used with other plaque-modifying interventions such as ELCA.

In this study, OCT examination demonstrated a reduction in plaque burden following ELCA. Post-procedural OCT images showed no significant residual stenosis. At one-year follow-up, OCT revealed a small soft eccentric plaque with mild positive remodelling. This represents a marked improvement compared with pre-procedural findings, where the patient had healed plaques that may predispose to plaque instability and rupture. Although this case report demonstrates encouraging imaging findings, it remains uncertain whether these improvements can be attributed solely to DCB therapy, given the combined use of ELCA and DCB treatment. Further studies are required to isolate the independent effects of DCB therapy on plaque morphology.

The findings from Funayama et al. 2021 suggest reductions in imaging-derived plaque measurements in both ACS and stable angina subgroups following DCB treatment. Intravascular imaging demonstrated maintained vessel patency with evidence of positive remodelling and reductions in plaque area following DCB therapy. These findings may have important clinical implications, suggesting that DCB is a promising treatment option in selected patients with coronary artery disease. It may also offer advantages over strategies such as DES or adjunctive plaque-modifying techniques such as ELCA, where a “leave-nothing-behind” strategy is desirable. However, larger studies are required to determine whether specific ACS subgroups benefit from revascularisation strategies [20].

Currently, multiple types of DCB devices are used in clinical practice, most of which utilise a paclitaxel-based coating. In this review, the SeQuent Please DCB (B. Braun, Germany) was most frequent, with only one study using a paclitaxel-coated balloon from Bingo™, (Dalian, China). In this study, 46 patients with 54 coronary lesions were treated with DCB therapy, resulting in late lumen enlargement in 74.1% of lesions. Additionally, mean plaque volume decreased from 53.8% to 47.8%, suggesting plaque regression following DCB treatment. The authors proposed that local delivery of paclitaxel to the vessel wall may promote plaque regression through antiproliferative effects. Paclitaxel distribution was enhanced when it was filtered and concentrated near the adventitial layer of the coronary arteries. The authors suggest that paclitaxel distribution within the vessel wall may influence plaque regression. Although this study was not included in the present review, due to the predominance of non-ACS patients, it provides important insight into how DCB therapy may influence plaque morphology.

In comparison, van Veelan et al. 2024 reported no significant change in overall plaque burden following DCB treatment, although a significant reduction in lipid-rich plaque content was observed. Specifically, the maximum lipid core burden index (maxLCB_4mm_) decreased from 431 (IQR 362–519) at baseline to 331 (IQR 206–461) at follow-up (*p* = 0.002). This suggests that DCB therapy may influence plaque composition even when overall plaque size remains relatively unchanged [19].

These findings also emphasise the importance of analysing lipid-related plaque characteristics in future studies, as changes in plaque lipid content may influence plaque stability and risk of rupture. Modification of lipid-rich plaque content may represent an additional mechanism through which DCB therapy reduce future MI risk.

Comparative studies by Zhang et al. 2022, Yamada et al. 2023, and Fukuoka et al. 2024, reported mixed results regarding the effects of DCB therapy on plaque morphology. In the study by Zhang et al. 2022, no significant differences in plaque burden reduction were observed between the DCB and DES groups. However, plaque burden decreased substantially within the DCB group, supporting the view that DCB performs comparably to DES while preserving the advantages of a leave-nothing-behind strategy [21].

Similarly, Yamada et al. 2023 reported that DCB and DES strategies demonstrated comparable safety and efficacy in the treatment of de novo coronary lesions, although DCB therapy was associated with significantly lower late lumen loss. This suggests that DCB therapy may promote better long-term lumen preservation. The authors proposed that paclitaxel delivery may explain late lumen enlargement in DCB-treated vessels [22].

Finally, Fukuoka et al. 2024 demonstrated that DCB-treated lesions were associated with lower plaque volume and reduced calcification scores compared with bailout stent lesions, supporting the feasibility of DCB in less complex plaque morphologies and selected ACS presentations. OCT imaging also demonstrated plaque rupture following DCB treatment, suggesting lesion modification during the procedure. Calcification represents a key structural component of atherosclerotic plaque, and differences in calcification deposition may influence procedural success and plaque modification during coronary intervention. These findings suggest that DCB therapy may influence plaque composition and structural characteristics, although further research is required to better understand the mechanisms underlying these effects [23].

### 4.1 Strengths and limitations

From the initial screening of 2550 records, only six studies met the inclusion criteria, highlighting the limited but emerging on evidence DCB and plaque morphology. Although some studies employed qualitative or thematic analyses, they reported imaging findings related to plaque morphology [18], [22], [24]. Qualitative analysis can provide insight into plaque characteristics. Furthermore, these studies highlighted the importance of evaluating additional plaque-related characteristics, including lipid core burden, calcification and late lumen enlargement, which are associated with plaque regression and vascular remodelling.

Another strength of this review is the inclusion of studies employing multiple intracoronary imaging modalities. This methodological flexibility allowed a broader analysis of plaque morphology across studies, although IVUS was the most used imaging modality. One study utilised combined IVUS-NIHRS [19]. QCA was not used in any of the included studies, as it primarily evaluates vessel diameter and luminal stenosis rather than providing detailed plaque morphology.

Imaging modalities provide detailed insight into vessel structure and plaque characteristics, allowing quantification of plaque area, volume and burden. Together, these modalities provide complementary information regarding plaque morphology and composition.

Despite these strengths, several limitations should be acknowledged. Firstly, few studies examined changes in plaque composition, with most studies focusing on plaque size or burden rather than biochemical or structural characteristics. Van Veelen et al. 2024 demonstrated a reduced lipid core burden following DCB treatment [19], while Fukuoka et al. 2024 reported lower calcification scores in DCB-treated lesions [23]. These findings suggest that DCB therapy may influence plaque composition, potentially affecting plaque stability and inflammatory activity. However, the limited studies assessing plaque composition make definitive conclusions difficult.

A potential future approach to address this limitation would be integrating advanced molecular techniques such as mass spectrometry-based lipidomic analysis, which could provide sensitive characterisation of lipid species within atherosclerotic plaques. When combined with intracoronary imaging, it may allow a comprehensive assessment of plaque composition before and after DCB therapy, offering insight into mechanisms underlying plaque modification.

Another limitation of this review is that only English-language studies were included, which may have excluded relevant research published in other languages. However, the PRISMA screening process excluded only one non-English paper (Chinese). All included studies utilised paclitaxel-based DCB technology in the available literature. This represents a limitation of current evidence base rather than the review methodology, as the inclusion criteria did not restrict the type of drug coating used in DCB devices. Further research investigating alternative technologies, such as sirolimus-coated balloons, is therefore warranted, as this would broaden the evidence base, allowing comparison of antiproliferative agents in relation to plaque modification.

Overall, limited studies have investigated the effects of DCB therapy on plaque morphology, with even fewer examining plaque composition. This represents an important area for future research, given the potential implications for plaque stabilisation and reduced cardiovascular events.

## 5 Conclusion

DCBs have been associated with reductions in plaque-related imaging parameters, including plaque area, volume and burden. However, emerging evidence suggests that DCB therapy may influence plaque composition, not solely plaque size. This highlights the need for further investigation, given limited current evidence.

Furthermore, additional research is required to understand the mechanisms of drug delivery from DCBs and how these influence plaque modification. Paclitaxel distribution within the vessel wall may play an important role in plaque regression [24]. Importantly, DCB technologies are heterogeneous, differing in drug type (e.g. paclitaxel or sirolimus), coating and excipients (such as urea, shellac or iopromide), and drug delivery system (matrix, crystalline or nanoparticle platforms). These device-specific differences may influence drug transfer efficiency and biological effects on the arterial wall [25].

Further studies are required to assess the clinical relevance of DCB therapy across ACS and stable angina populations. Future investigations should evaluate plaque composition, particularly lipid-related characteristics, to determine whether DCB therapy contributes to plaque stabilisation beyond plaque size reduction. More detailed plaque assessment may be achieved using mass spectrometry-based lipidomic analysis, combined with intracoronary imaging modalities.

By clarifying whether DCB therapy is associated with meaningful changes in plaque composition and lipid-related characteristics, future studies may identify predictors of treatment response and improve patient selection for DCB-based interventions. Ultimately, this may open opportunities for lipid-targeted adjunctive therapies, enhancing vessel healing, reducing restenosis, and improving long-term clinical outcomes in coronary artery disease.

## CRediT authorship contribution statement

**Kavithra Harihararajah:** Data curation, Investigation, Methodology, Writing – original draft, Writing – review & editing. **Zaynah Nawaz:** Data curation, Formal analysis, Investigation, Visualization, Writing – original draft. **Laiba Qadir:** Data curation, Formal analysis, Investigation, Visualization, Writing – original draft, Writing – review & editing. **Shakil Ahmad:** Investigation, Methodology, Project administration, Supervision, Validation, Writing – review & editing. **Derek Connolly:** Funding acquisition, Investigation, Methodology, Project administration, Resources, Supervision, Validation, Writing – review & editing. **Irundika H.K. Dias:** Conceptualization, Funding acquisition, Investigation, Methodology, Project administration, Resources, Supervision, Visualization, Writing – review & editing. **Mohammed Shamim Rahman:** Conceptualization, Data curation, Funding acquisition, Investigation, Methodology, Project administration, Resources, Supervision, Validation, Visualization, Writing – original draft, Writing – review & editing.

## Ethical statement

This article is a systematic review based solely on previously published studies and does not include the collection or analysis of original data.

Therefore, institutional ethical approval was not required. No human participants, human tissue, or identifiable personal data were involved in the preparation of this manuscript.

## Funding

This work was supported by the Listers Cardiovascular Research Group (Aston University and Sandwell and West Birmingham NHS Trust). No external funding was received.

## Declaration of competing interest

The authors declare no conflicts of interest.

## Data Availability

All data analysed in this study are derived from previously published articles, which are cited within the manuscript. No new datasets were generated.
